# Zungennekrose als klinische Manifestation einer Riesenzellarteriitis

**DOI:** 10.1007/s00108-023-01607-w

**Published:** 2023-10-20

**Authors:** Alexander Pfeil, Tobias Hoffmann, Martin Freesmeyer, Peter Oelzner, Gunter Wolf

**Affiliations:** 1 Universitätsklinikum Jena, Klinik für Innere Medizin III, Friedrich-Schiller-Universität Jena, Am Klinikum 1, 07747 Jena, Deutschland; 2 Universitätsklinikum Jena, Klinik für Nuklearmedizin, Friedrich-Schiller-Universität Jena, Jena, Deutschland

**Keywords:** Riesenzellarteriitis, Zungennekrose, Sonographie, Positronenemissionstomographie/Computertomographie, Glukokortikoide, Giant cell arteritis, Tongue necrosis, Ultrasound, Positron emission tomography/computed tomography, Corticosteroids

## Abstract

Ein 83-jähriger Patient stellte sich aufgrund einer seit drei Wochen bestehenden Schwellung der Zunge vor. Die Zungenschwellung ging mit Schmerzen beim Essen, gelblichen Plaques sowie einer gräulich-braunen Läsion im vorderen Abschnitt der Zunge einher. Zusätzlich waren ein plötzlicher Sehverlust auf dem linken Auge und temporale Kopfschmerzen seit drei Tagen zu verzeichnen. Aufgrund eines paraklinisch erhöhten C‑reaktiven Proteins und der bestehenden Symptomatik wurden eine Sonographie der supraaortalen Arterien und auch eine Positronenemissionstomographie/Computertomographie durchgeführt. Es zeigten sich in der Bildgebung eine Entzündung der großen Arterien sowie ein Halophänomen in der Sonographie der A. temporalis. Somit konnte die Diagnose einer Riesenzellarteriitis mit Zungennekrose gestellt werden. Eine immunsuppressive Therapie mit Glukokortikoiden wurde eingeleitet. Die Zungennekrose stellt eine seltene Manifestation einer Riesenzellarteriitis dar, welche einer umgehenden immunsuppressiven Therapie bedarf, um weitere Folgeschäden, wie vollständige Nekrose und Superinfektion bis zur Zungenamputation, zu vermeiden.

## Abstract (en)

An 83-year-old male patient presented due to a 3-week history of swelling of the tongue with tongue pain on eating, yellowish plaques, and a gray-brown lesion in the anterior portion of the tongue. Sudden loss of vision in the left eye and temporal headache occurred 3 days before presentation. Due to elevated C‑reactive protein, sonography of the supraaortic arteries as well as positron emission tomography/computed tomography was performed. Imaging revealed inflammation of the great arteries as well as a halo sign on ultrasound of the temporal artery. Thus, a diagnosis of giant cell arteritis with necrosis of the tongue was made. Immunosuppressive therapy with glucocorticoids was initiated. Necrosis of the tongue is a rare manifestation of giant cell arteritis that requires immediate immunosuppressive therapy to prevent further complications to the tongue (e.g., complete necrosis of the tongue, superinfection, tongue amputation).

## Anamnese

Ein 83-jähriger Mann wurde aufgrund einer seit drei Wochen bestehenden Schwellung der Zunge mit Zungenschmerzen beim Essen vorgestellt. Des Weiteren berichtete der Patient über eine gräulich-braune Verfärbung der Zunge. Zusätzlich waren ein plötzlicher Sehverlust auf dem linken Auge und temporale Kopfschmerzen seit drei Tagen zu verzeichnen.

## Untersuchung

**Figure 1:**
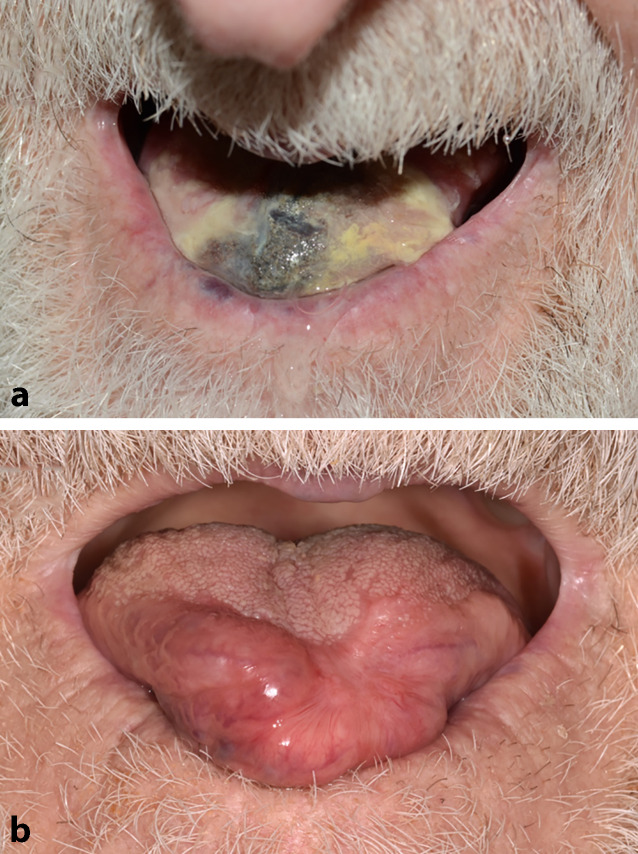

Die Inspektion der Zunge ergab ein diffuses Ödem mit gelblichen Plaques sowie eine gräulich-braune Läsion im vorderen Abschnitt der Zunge (Abb. 1a). Die körperliche Untersuchung zeigte zudem eine verhärtete linke A. temporalis.

## Diagnostik

### Labor

Laborchemisch wurde ein deutlich erhöhtes C‑reaktives Protein von 73,0 mg/l (Referenz: < 2,0 mg/l) bei normaler Leukozytenzahl nachgewiesen. Die weiteren laborchemischen und immunologischen Untersuchungen erbrachten keinen richtungsweisenden Befund.

### Bildgebung

**Figure 2:**
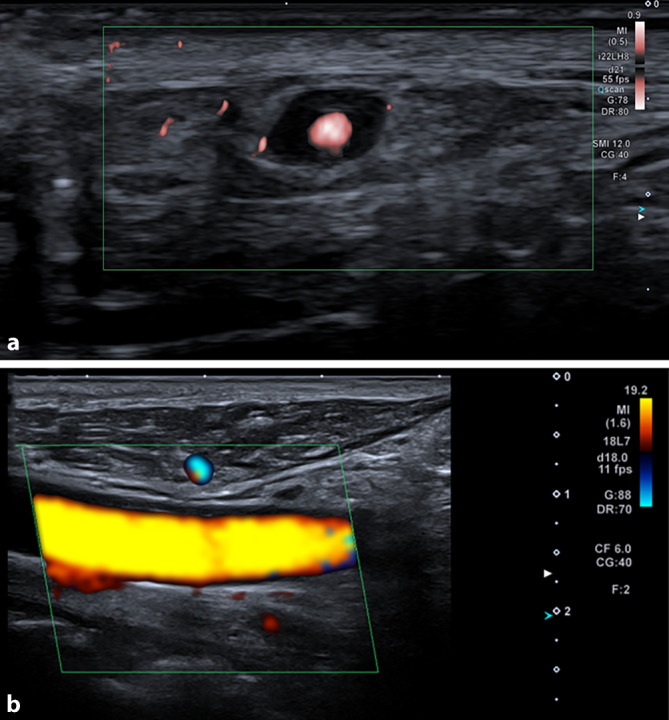

**Figure 3:**
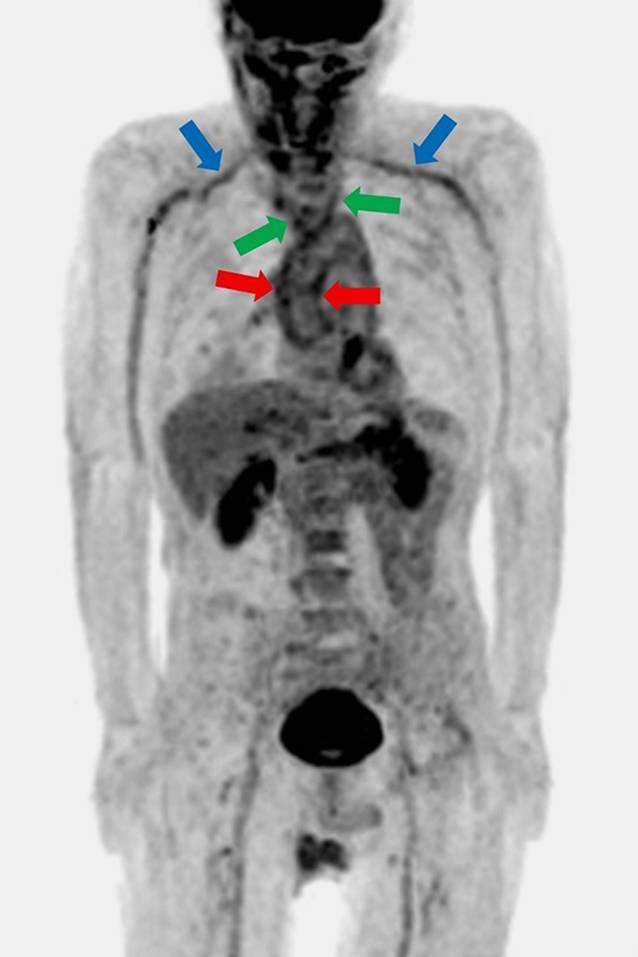

In der farbcodierten Dopplersonographie konnten eine nicht komprimierbare zirkuläre Wandschwellung (Halozeichen) der Temporalarterien (Abb. 2a) sowie ein entzündliches Gefäßwandödem der A. carotis communis, A. carotis interna und externa (Abb. 2b) abgebildet werden. In der ^18^F‑Fluordesoxyglukose-Ganzkörper-Positronenemissionstomographie/Computertomographie wurde zusätzlich eine Gefäßwandentzündung der Aorta, der A. axillaris, der A. subclavia und der A. vertebralis dargestellt (Abb. 3).

## Diagnose

- Auf der Grundlage der klinischen Präsentation, der laborchemischen Entzündungsparameter sowie der Bildgebung wurde die Diagnose einer Riesenzellarteriitis mit einer Zungennekrose gestellt.

## Therapie und Verlauf

Es erfolgten die Einleitung eines Methylprednisolonpulses (Methylprednisolon 500 mg i.v. über drei Tage) und folgend eine leitliniengerechte Glukokortikoidtherapie [1]. Hierunter kam es zu einer Normalisierung des C‑reaktiven Proteins. Aufgrund der nicht vollständigen Rückbildung der Zungennekrose erfolgte die Durchführung einer Nekrosektomie. Nachfolgend kam es zur narbigen Ausheilung des Wunddefekts (Abb. 1b). Im Verlauf kam es zu einer geringen Verbesserung des Sehvermögens, ohne dass das Ausgangsniveau wieder erreicht werden konnte. Unter der Therapie zeigten die temporalen Kopfschmerzen eine vollständige Regredienz.

## Diskussion

Die Riesenzellarteriitis (RZA) ist eine entzündlich-immunologisch vermittelte Erkrankung der mittleren sowie großen Arterien mit einer variablen Gefäßbeteiligung [2]. Das Erkrankungsbild kann in eine kranielle bzw. extrakranielle Form unterteilt werden [1]. Der kraniellen RZA wird die Arteriitis temporalis, aber auch die RZA der Aa. facialis, occipitalis, lingualis und der intrakraniellen Arterien zugeordnet [3, 4]. Weiterführend ist die extrakranielle Form der RZA durch eine Beteiligung der Aorta und deren Gefäßabgänge (Aa. axillares, carotides, subclaviae, vertebrales, femorales) gekennzeichnet [3–5]. Aktuelle Daten bezüglich der Gefäßbeteiligung bei einer RZA zeigen folgende Verteilung: 48 % kranielle RZA, 21 % extrakranielle RZA und 32 % gemischte Form bestehend aus einer kraniellen und extrakraniellen RZA [3]. In einer weiterführenden Studie von Malich et al. wurde eine isolierte Beteiligung der Aorta und des Truncus brachiocephalicus bei 20 % der RZA-Patienten in der PET/CT nachgewiesen [5].

Systemische Symptome wie Fieber, Nachtschweiß, Gewichtsverlust, Husten und Fatigue treten bei einer extrakraniellen Manifestation auf, wobei die Symptomatik einer Polymyalgia rheumatica bei beiden Unterformen der RZA vorhanden sein kann [5, 6].

Klinische Zeichen einer kraniellen RZA sind Kopfschmerzen (insbesondere temporale Kopfschmerzen), Kauschmerzen sowie eine Sehverschlechterung bis hin zur Amaurosis fugax bei einem entzündlichen Verschluss der retinalen Arterien [5, 6]. Des Weiteren kann in sehr seltenen Fällen der komplette Verschluss der A. temporalis mit einer Kopfhautnekrose verbunden sein [7]. Demgegenüber ist eine intrakranielle RZA mit zerebrovaskulären ischämischen Ereignissen assoziiert [8]. Das Auftreten einer Zungennekrose bei einer RZA wird nur in wenigen Fällen beschrieben [9]. Die Zungennekrose ist auf eine entzündliche Beteiligung der A. lingualis zurückzuführen, welche wie auch die A. temporalis aus der A. carotis externa entspringt.

Eine histologische Sicherung der Entzündung der A. lingualis wie auch eine bildgebende Darstellung ist nicht möglich. Aus diesem Grund ist der bildgebende Nachweis einer RZA auf einen in der Bildgebung zugänglichen Gefäßabschnitt (z. B. A. temporalis, A. carotis externa, interna oder communis) zu fokussieren. Als bildgebende Techniken können die Sonographie, die Magnetresonanztomographie oder die Positronenemissionstomographie/Computertomographie (PET/CT) eingesetzt werden [10], wobei eine bildgebende Darstellung der A. temporalis durch die limitierte Ortsauflösung der PET/CT nur in begrenztem Umfang möglich ist [11].

Die primäre Therapie einer Zungennekrose fokussiert auf die antiinflammatorische Therapie der RZA. Für die RZA-Therapie sollten primär Glukokortikoide (40–60 mg täglich) eingesetzt werden. Bei Sehstörungen ist ein Methylprednisolonpuls (500 mg bis 1000 mg über drei bis fünf Tage) zu initiieren [1]. Die Glukokortikoidtherapie ist unverzüglich einzuleiten, um schwerwiegende Komplikationen der Vaskulitis wie z. B. eine ausgedehnte Nekrose der Zunge und eine hieraus resultierende Notwendigkeit einer Zungenamputation zu vermeiden [12].

Bei einem Rezidiv erfolgen die Erhöhung der Glukokortikoiddosis und die Hinzunahme einer weiterführenden immunsuppressiven Therapie mit Tocilizumab (Antikörper gegen den Interleukin-6-Rezeptor) oder Methotrexat [1]. Unter der immunsuppressiven Therapie sollte eine Abheilung der Zungennekrose erfolgen.

Eine chirurgische Therapie der Zungennekrose ist erst bei supprimierter Entzündung und fehlender Abheilung oder zur Entfernung von residualem nekrotischem Gewebe zu diskutieren. Im Stadium der floriden RZA sollte kein operativer Eingriff zur primären Therapie durchgeführt werden, da aufgrund der entzündeten Arterien keine ausreichende Wundheilung zu erwarten ist und die chirurgische Therapie kein adäquates therapeutisches Verfahren einer RZA darstellt.

Differenzialdiagnostisch sollten als weitere Ursachen für eine Zungennekrose Kleingefäßvaskulitiden, Malignome, medikamentöse Nebenwirkungen bei ergotaminhaltigen Präparaten, Strahlentherapie, kardiovaskuläre Ursachen (z. B. Embolie) und Infektionen (z. B. Herpes) ausgeschlossen werden [12].

Nach eingeleiteter Glukokortikoidtherapie war aufgrund der antiinflammatorischen Wirkung eine verbesserte Durchblutung der Zunge gegeben, sodass eine partielle Abheilung der Zunge erzielt werden konnte. Insbesondere der Einsatz von Glukokortikoiden ist mit einer signifikanten und schnellen Abnahme des entzündlichen Gefäßwandödems verbunden [13], sodass eine Reperfusion des Gewebes ermöglicht und das Ausmaß eines irreversiblen Gewebeschadens begrenzt werden kann.

Zusammenfassend stellt die Zungennekrose eine selten auftretende Komplikation einer Riesenzellarteriitis dar, welche auf eine entzündliche Mitbeteiligung der A. lingualis zurückzuführen ist. Eine histologische Sicherung oder bildgebende Darstellung der Vaskulitis der A. lingualis ist nicht möglich, sodass die bildgebende Evaluation über eine systematische Darstellung der großen Arterien erfolgen sollte. Die immunsuppressive Therapie mit Glukokortikoiden stellt die primäre Therapie der Wahl dar.

## Fazit für die Praxis

- Die Zungennekrose stellt eine seltene klinische Manifestation einer Riesenzellarteriitis dar.
- Eine Beurteilung der entzündlichen Manifestation der RZA wird über eine Bildgebung der großen Arterien vorgenommen.
- Therapeutisch werden Glukokortikoide und bei einem Therapieversagen Tocilizumab (Antikörper gegen den Interleukin-6-Rezeptor) oder Methotrexat eingesetzt.

## Abkürzungen

PET/CT: Positronenemissionstomographie/Computertomographie
RZA: Riesenzellarteriitis
